# Cost-utility analysis of eptinezumab for migraine prevention in Taiwan

**DOI:** 10.1186/s13561-025-00711-x

**Published:** 2025-12-28

**Authors:** Cheng-Shen Chan, Tzu-Yao Huang, Wei-Hsuan Tseng, Tsung-Kun Lin, Fu-Chi Yang, Yi Liu, Yuan-Zhen Ruan, Ping-Hsuan Hsieh

**Affiliations:** 1 College of Pharmacy, National Defense Medical University, No. 161, Sec. 6, Minquan E. Rd., Neihu Dist., Taipei, 114202 Taiwan; 2 Department of Pharmacy, Tri-Service General Hospital Songshan Branch, Taipei, Taiwan; 3 Department of Pharmacy, Tri-Service General Hospital, National Defense Medical University, Taipei, Taiwan; 4 The Dartmouth Institute for Health Policy and Clinical Practice, Geisel School of Medicine at Dartmouth, Hanover, NH USA; 5 College of Medicine, National Defense Medical University, Taipei, Taiwan; 6 Department of Neurology, Tri-Service General Hospital, National Defense Medical University, Taipei, Taiwan

**Keywords:** Eptinezumab, CGRP, Migraine, Cost-utility analysis, Economic evaluation

## Abstract

**Aim:**

Migraine is a neurological disorder prevalent in Taiwan, affecting millions of individuals and imposing a substantial burden on both quality of life and societal productivity. Calcitonin gene-related peptide inhibitors, such as eptinezumab, represent a major advancement in migraine prevention; however, their high cost and the lack of local economic evaluations warrant further study. This research aims to assess the cost-effectiveness of eptinezumab, compared with placebo, for migraine prevention in Taiwan.

**Methods:**

A Markov model with a six-month time horizon was developed to evaluate the cost-effectiveness of eptinezumab versus placebo. The analysis was conducted from a health payer’s perspective, incorporating clinical and economic inputs from clinical trials and the literature. Patients were categorized into six health states based on monthly migraine days. Outcomes were expressed as incremental costs per quality-adjusted life year (QALY) gained. Incremental cost-effectiveness ratios (ICERs) were estimated using a Monte Carlo simulation with 10,000 iterations, alongside deterministic and probabilistic sensitivity analyses to evaluate uncertainty. For interpretation, results were compared with Taiwan’s GDP per capita ($32,327 per QALY), and exploratory analyses also considered a threshold of three times GDP per capita ($96,981).

**Results:**

Over six months, the total cost for patients receiving eptinezumab was $4,461, compared with $1,065 for placebo, with corresponding QALYs of 0.35 and 0.31, respectively. This yielded an ICER of $73,929 per QALY gained. Deterministic sensitivity analysis identified utility values for patients with varying migraine frequency as the most influential parameter, increasing the ICER to approximately $105,000 when varied, which may exceed the upper WTP threshold. Probabilistic sensitivity analysis showed that eptinezumab was cost-effective in 98% of iterations at the exploratory threshold of three times GDP per capita.

**Conclusion:**

Eptinezumab was likely cost-effective for migraine prevention in Taiwan, with utility values associated with migraine frequency identified as the key driver, highlighting the need for locally relevant quality-of-life data in future evaluations.

**Supplementary Information:**

The online version contains supplementary material available at 10.1186/s13561-025-00711-x.

## Highlights

• This study is the first to evaluate the cost-effectiveness of eptinezumab for migraine prevention in Taiwan.

• Eptinezumab demonstrated an incremental cost-effectiveness ratio of $73,929 per QALY gained and is considered cost-effective in the context.

• Utility values associated with different levels of migraine frequency were the most influential factor affecting the model outcomes.

**Supplementary Information:**

The online version contains supplementary material available at 10.1186/s13561-025-00711-x.

## Introduction

Migraine, a widespread and debilitating neurological disorder, affects approximately 12% of the global population, with a higher prevalence among women [1]. According to the Global Burden of Disease Study, migraine is ranked as the second leading cause of disability worldwide [2]. In Taiwan, the prevalence of migraine is currently 9.1% [3]. The epidemiological pattern reveals a female predominance, with women being 2–3 times more likely to experience migraine than men; this is consistent with global trends. The International Headache Society classifies migraines into two categories: episodic migraine (EM), characterized by headaches occurring 0–14 days per month, and chronic migraine (CM), characterized by headaches occurring 15 or more days per month [4]. A previous study estimated that migraine-related absenteeism leads to a loss of approximately 3.7 million workdays annually, resulting in an economic burden of NT$4.6 billion [5]. These findings underscore the substantial impact of migraines on both individual quality of life and broader health insurance costs.

Preventive treatment is recommended for patients experiencing headaches at least four days per month, those who have not responded to or cannot tolerate acute medications, or those suffering from unusual migraine types [6]. Traditionally, oral medications such as β-blockers, calcium channel blockers, antiepileptics, and antidepressants have served as the mainstay of migraine prevention. However, many of these medications are prescribed off-label, often resulting in poor tolerability or inadequate efficacy. Consequently, adherence to preventive regimens is often low, further exacerbating the burden of disease [7, 8]. Moreover, preventive treatment typically requires a minimum duration of six months, necessitating sustained patient adherence. During this period, challenges such as unmet expectations for rapid symptom relief, side effects, and difficulties in maintaining adherence may arise [9]. The ongoing risk of medication overuse can also lead to medication-overuse headaches, even among patients who continue treatment [10]. 

In contrast to traditional preventive therapies, calcitonin gene-related peptide (CGRP) antagonists represent the first class of medications developed specifically for migraine prevention, rather than being repurposed from other indications. This mechanism-based approach targets the trigeminal pain system [11] and offers a migraine-specific therapeutic option with favorable tolerability profiles [12–16]. CGRP inhibitors signify a paradigm shift in migraine management, addressing the underlying pathophysiology rather than solely managing symptoms [16]. Among these agents, eptinezumab, which was approved by the U.S. Food and Drug Administration in 2020, has shown particular promise for migraine prevention [17–19]. Notably, its therapeutic effects can be observed as early as one day after administration [20], demonstrating a faster onset of action than other CGRP inhibitors [21]. Clinically, eptinezumab is distinct from other CGRP inhibitors in that it is administered intravenously once every three months, potentially offering a more convenient treatment schedule.

Despite evidence supporting the clinical efficacy of CGRP inhibitors, their high cost remains a significant concern for healthcare systems and payers [22]. Given the limited data on the cost-effectiveness of eptinezumab, particularly in Asian populations, and the growing interest in its potential inclusion in Taiwan’s National Health Insurance (NHI) program, a robust evaluation of its economic value is warranted. This study addresses this gap by assessing the cost-effectiveness of eptinezumab for migraine prevention in Taiwan and providing insights to support healthcare decision-making.

## Methods

A cost-utility analysis (CUA) was conducted over a six-month time horizon to estimate the incremental cost per quality-adjusted life year (QALY) gained with eptinezumab compared with placebo, using 12-week cycles to match the dosing schedules in the PROMISE and DELIVER trials [17–19]. The analysis adopted a health payer’s perspective, focusing on direct medical costs, including acute treatment, hospitalization, outpatient visits, and the cost of eptinezumab. Although migraine is a chronic condition that may persist for decades, a six-month time horizon was chosen to align with the dosing schedules and primary efficacy endpoints of the PROMISE and DELIVER trials. This conservative approach minimizes uncertainty from extrapolating treatment effects beyond available clinical data. Moreover, this timeframe captures a complete treatment cycle of eptinezumab, administered quarterly, allowing assessment of sustained efficacy over multiple doses [23]. Efficacy data for patient transitions were derived from clinical trials. This study adheres to the Consolidated Health Economic Evaluation Reporting Standards (CHEERS) 2022 checklist to ensure transparent reporting [24]. 

### Population

The modeled population included adults aged 18–75 years who had received either 100 mg of eptinezumab or a placebo, as documented in the PROMISE and DELIVER trials [17–19]. Eligible patients must have received a migraine diagnosis by the age of 50 years, experienced migraines for at least 12 months, and reported at least four monthly migraine days (MMDs). Patients with EM and CM were included, with EM defined as experiencing 0–14 MMDs and CM defined as experiencing 15 or more MMDs. Individuals with confounding or clinically significant pain syndromes, temporomandibular disorders, or uncontrolled or untreated psychiatric conditions were excluded.

Patient characteristics were drawn from the clinical trials. Age at entry was sampled using the reported means and standard deviations, which ranged from 40.0 to 44.6 years across studies. The cohort was predominantly female (approximately 78%). Baseline monthly migraine days were sampled by migraine type using beta distributions fitted to trial data, with PROMISE-1 reporting 10.0 days, PROMISE-2 reporting 20.4 days, and DELIVER reporting 14.5 days. A weighted average across the three trials was applied in the model. Sampling was conducted independently of age and gender to ensure the simulated cohort reflected the key characteristics of the trial populations. Additional details are provided in the Supplemental Materials.

### Model structure

**Fig. 1:**
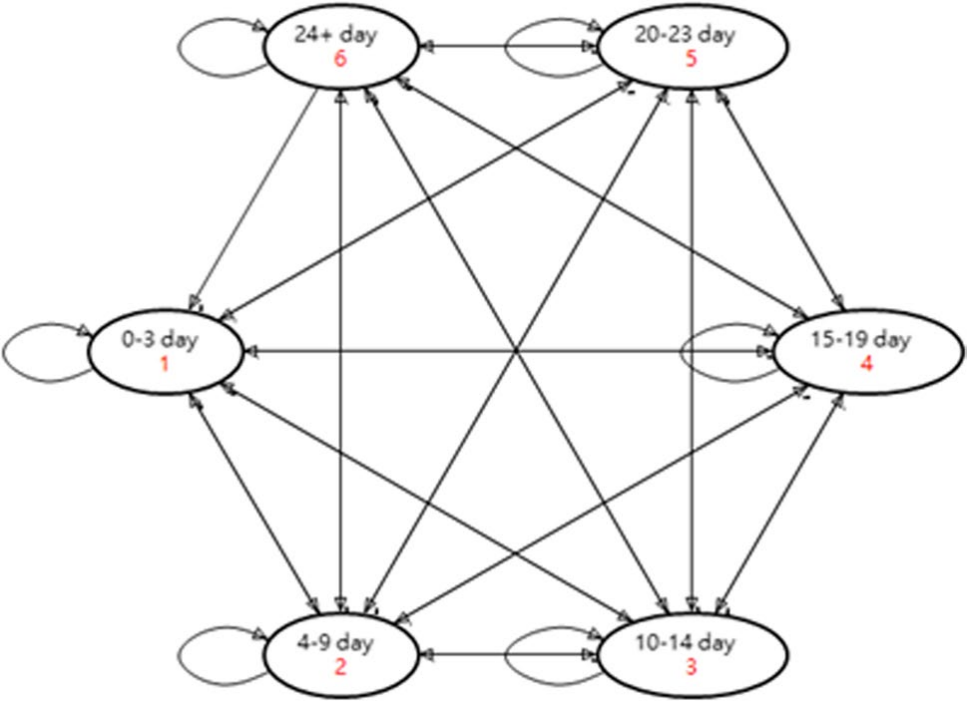
Markov model representing transitions between MMD states. Note: Arrows represent allowed transitions between states, reflecting changes in migraine frequency within a given model cycle

The analysis employed a Markov model adapted from prior literature [25], comprising six health states classified by the number of MMDs (Fig. 1). Patients with EM were categorized into three groups: 0–3, 4–9, and 10–14 MMDs, while those with CM were categorized into 15–19, 20–23, and ≥ 24 MMDs. The baseline distribution of patients across these health states was based on data from the PROMISE-1 and PROMISE-2 trials. Given the relatively young mean age of the population (42 years) and the short time horizon, mortality was considered negligible and excluded from the model.

### Treatment effect

Patients were assumed to transition between health states at the beginning of each 12-week cycle, with transition probabilities estimated from clinical trial data. Probabilities were calculated using bootstrapping with 1,000 resamples to generate mean values and standard errors (Table 1). As the clinical trials reported a medication discontinuation rate of less than 1% due to side effects, [17–19] discontinuation was not incorporated into the model (Table 2). Transition probabilities were normalized so their sum equaled 100% within each cycle. Additional details regarding the sampling distributions are available in the Supplemental Methods.

**Table 1:** Transition probabilities among health States based on monthly migraine day ranges from the PROMISE-1, PROMISE-2, and DELIVER trials

Eptinezumab (placebo)	0–3 days	4–9 days	10–14 days	15–19 days	20–24 days	24+ days
0–3 days	1 (1)	0.56 (0.29)	0.05 (0.01)	0.01 (0)	0 (0)	0 (0)
4–9 days	0 (0)	0.44 (0.71)	0.74 (0.53)	0.28 (0.08)	0.04 (0)	0 (0)
10–14 days	0 (0)	0 (0)	0.21 (0.46)	0.61 (0.61)	0.40 (0.16)	0.08 (0.01)
15–19 days	0 (0)	0 (0)	0 (0)	0.09 (0.31)	0.53 (0.64)	0.48 (0.27)
20–24 days	0 (0)	0 (0)	0 (0)	0 (0)	0.03 (0.20)	0.36 (0.50)
24 + days	0 (0)	0 (0)	0 (0)	0 (0)	0 (0)	0.09 (0.22)

Minor discrepancies in the sum of transition probabilities within columns are due to rounding, and therefore the total may not equal exactly 1

**Table 2:** Model inputs based on cost estimates and utility data

Description	Estimate (SD)	Distribution model
Utility values for eptinezumab and placebo by monthly migraine day range		
0–3 days	0.778 (0.010) [0.707 (0.010)]	Beta
4–9 days	0.732 (0.015) [0.661 (0.015)]	Beta
10–14 days	0.681 (0.012) [0.611 (0.013)]	Beta
15–19 days	0.635 (0.013) [0.565 (0.012)]	Beta
20–24 days	0.595 (0.010) [0.524 (0.010)]	Beta
24 + days	0.553 (0.012) [0.483 (0.013)]	Beta
Cost of eptinezumab (USD)		
Eptinezumab	1,708	Uniform
Health resources utilization (USD)		
MRI (without contrast)	195.9	Gamma
CT (without contrast)	114.5	Gamma
ECG	4.5	Gamma
Skull X-ray film (including each view of skull film)	6.0	Gamma
Blood test	6.0	Gamma
Botulinum toxin injection	110.4	Gamma
Transcutaneous electrical nerve stimulator	9.6	Gamma
Occipital nerve block	0.7 (0.4)	Gamma
Acute medications	394.9 (197.5)	Gamma
Prevention medications	37.0 (18.5)	Gamma
Cost of patients with CM (USD)		
Acute medications	270.0 (135.0)	Gamma
Prevention medications	37.0 (18.5)	Gamma
Cost of patients with EM (USD)		
Acute medications	184.0 (92.0)	Gamma
Prevention medications	27.4 (13.7)	Gamma

*Abbreviations*: *MRI* Magnetic resonance imaging, *CM* Chronic migraine, *CT* Computed tomography, *ECG* Electrocardiogram, *EM* Episodic migraine

^1^Utility data obtained from Wang et al. [30]

^2^Cost of drug and administration derived from National Institutes of Health .Costs for diagnostic tests and procedures were obtained from the Taiwan National Health Insurance database, and probabilities were derived from Wang et al. [30]

### Health-related quality-of-life (HRQoL)

Due to the absence of HRQoL data specific to eptinezumab in Taiwan, utility values were derived from a study involving Japanese and Korean patients treated with fremanezumab [27]. A beta regression model was used to estimate utilities as a function of monthly migraine days and patient characteristics. Wang et al. (2023) reported that raw EQ-5D-5 L values exhibited a narrow response range and were insufficiently sensitive to detect meaningful HRQoL changes in migraine populations. By contrast, MSQ-derived EQ-5D-3 L values demonstrated greater responsiveness to changes in migraine frequency and were therefore considered more appropriate [28]. These utilities were derived from Asian patients in fremanezumab trials and were deemed transferable to the cohort modeled in this analysis.

### Healthcare resource use and costs

The cost of eptinezumab was set at $1,708 per dose. Patients in the placebo group incurred only administrative costs. Estimates of migraine-related healthcare utilization, including costs of outpatient visits, emergency room visits, inpatient admissions, and medications, were obtained from a retrospective longitudinal study using data from the Taiwan National Health Insurance Research Database [29]. Costs related to diagnostic procedures (e.g., MRI, CT, ECG, X-rays, and blood tests) and treatment interventions (e.g., botulinum toxin injections, transcutaneous electrical nerve stimulation, and nerve blocks) were also included [26, 30]. All unit costs were based on the 2023 reimbursement schedule published by Taiwan’s National Health Insurance Administration, as detailed in Supplementary Table 1.

Both costs and QALYs were discounted at an annual rate of 3% in the base-case analysis. All monetary values were converted to 2024 U.S. dollars.

### Sensitivity analyses

Sensitivity analyses were conducted to address parameter uncertainty. One-way sensitivity analysis varied treatment outcomes and utility estimates by ± 10% and cost parameters by ± 20%. For interpretation, we defined one-times Taiwan’s GDP per capita as the benchmark for highly cost-effective interventions and three-times GDP per capita as the primary threshold for cost-effectiveness, consistent with commonly accepted practices in the absence of an official willingness-to-pay standard. Probabilistic sensitivity analysis was performed using 10,000 Monte Carlo simulations to estimate the distribution of incremental cost-effectiveness ratios (ICERs) and to generate cost-effectiveness acceptability curves. All analyses were conducted in TreeAge Pro 2024.

## Results

### Base-case analysis

In the base case analysis, treatment with eptinezumab reduced monthly migraine days (MMDs) by 5.8 days over six months (standard deviation [SD] 1.8), compared with a reduction of 3.8 days (SD 1.7) for placebo. This corresponded to a mean difference of 2.0 days between groups. The average total cost was $4,461 for patients receiving eptinezumab and $1,065 for those receiving placebo, resulting in an incremental cost of $3,396. The corresponding QALYs were 0.35 for the eptinezumab group and 0.31 for the placebo group, yielding a QALY gain of 0.05. As shown in Table 3, the ICER was $73,929 per QALY gained. When compared with Taiwan’s GDP per capita ($32,327 per QALY) and with an exploratory threshold of three times GDP per capita ($96,981), eptinezumab may be considered cost-effective.

**Table 3:** Results of cost (USD), QALY, and ICER

	Mean Cost	Incremental Cost	Mean QALYs	Incremental QALYs	ICER
Eptinezumab	$4,461	$3,396	0.35	0.05	$73,929
Placebo	$1,065		0.31		

Due to rounding of the cost and QALY values shown in the table, directly dividing 3,396 by 0.05 does not yield exactly 73,929

*Abbreviations*: *ICER* Incremental cost-effectiveness ratio, *QALYs* Quality-adjusted life years

### Sensitivity analyses

**Fig. 2:**
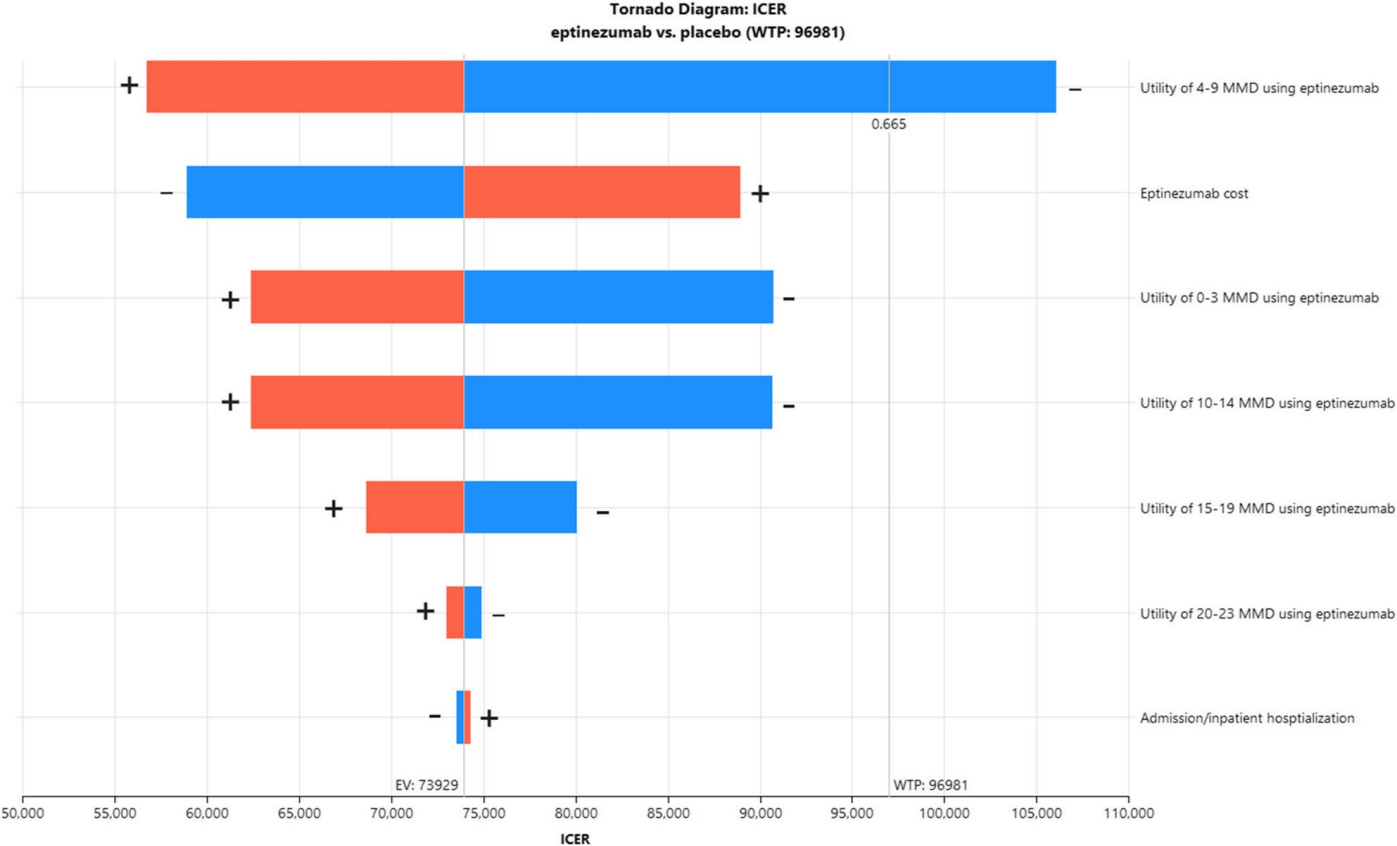
Tornado diagram of key parameters and their impact on the incremental cost-effectiveness ratio when varied. Note: Orange: Upper bound; Blue: Lower bound. Abbreviations: ICER – incremental cost-effectiveness ratio; MMD – monthly migraine days

A one-way sensitivity analysis (Fig. 2) showed that variations in utility values across migraine frequency categories (0–3, 4–9, 10–14, and 15–19 MMDs) had the greatest impact on the ICER. Adjusting the utility for patients with 4–9 MMDs in the eptinezumab group increased the ICER to approximately $105,000, which was the only scenario in the deterministic sensitivity analysis where the ICER exceeded the WTP threshold. Changes in transition probabilities between migraine health states by ± 10% had minimal effect, resulting in ICER differences of less than $1,000. When direct medical and drug acquisition costs were varied by ± 20%, only changes in drug costs had a notable impact, increasing the ICER to about $89,000. Overall, in most scenarios tested, the ICER remained below the GDP-based benchmarks, suggesting that the cost-effectiveness of eptinezumab was generally robust to parameter uncertainty.

**Fig. 3:**
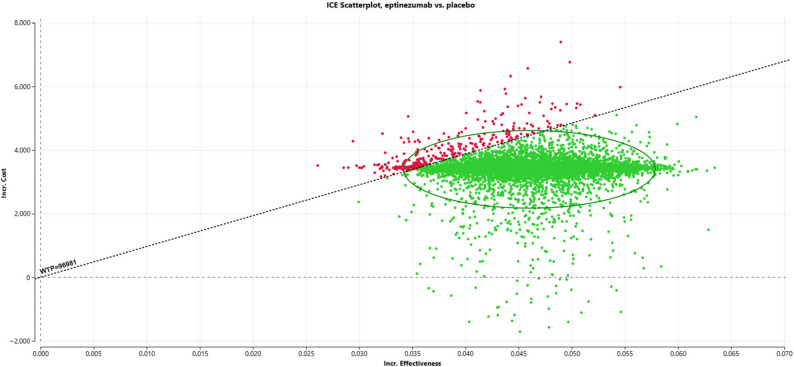
Cost-effectiveness scatterplot comparing eptinezumab versus placebo. Note: Green dots represent cost-effective outcomes; red dots indicate non-cost-effective outcomes. The slope represents willingness-to-pay, with a threshold set at three times Taiwan's per capita gross domestic product of $96,981

**Fig. 4:**
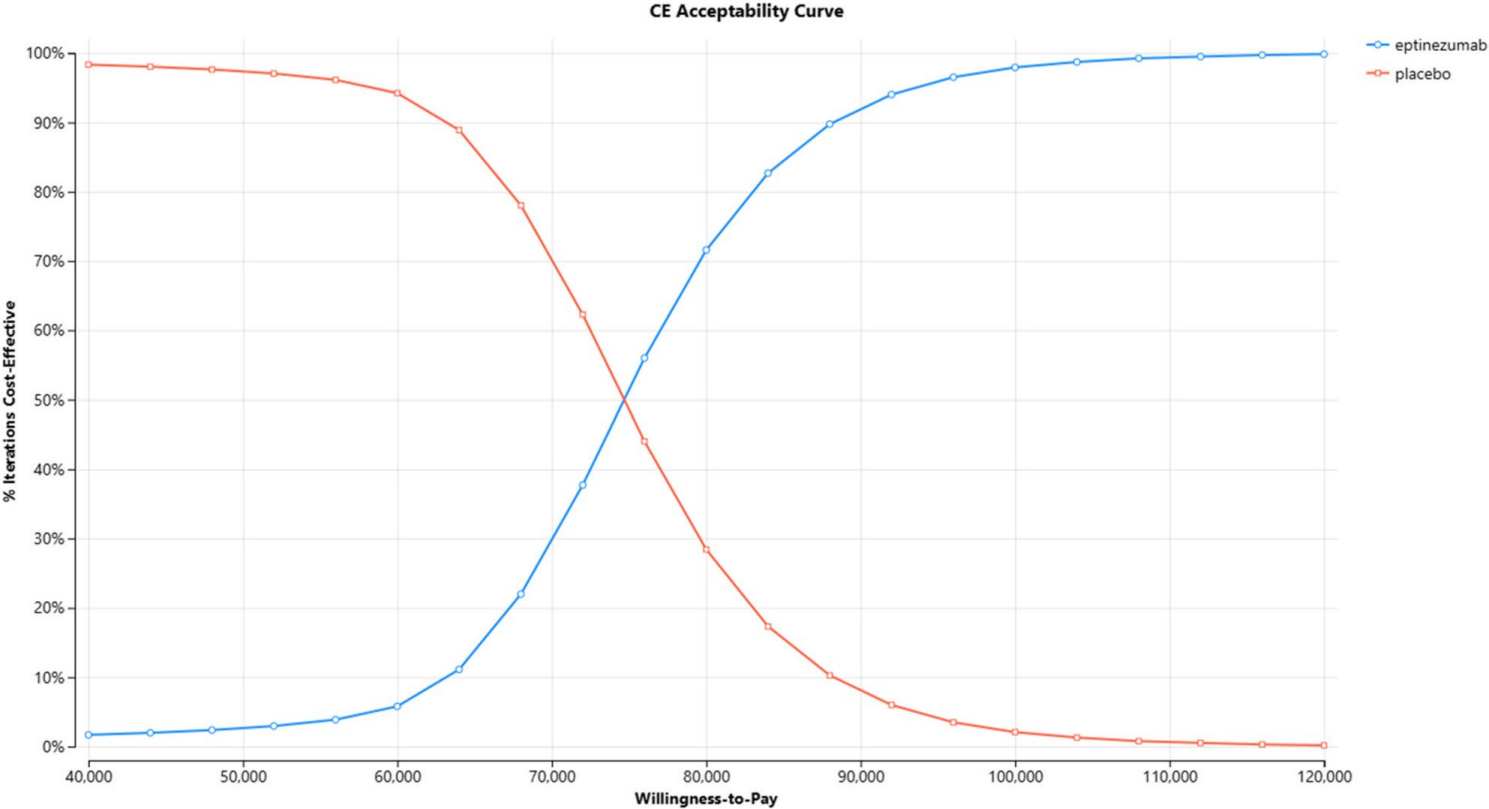
Cost-effectiveness acceptability curve comparing eptinezumab versus placebo. Note: The blue line represents the probability of eptinezumab being cost-effective; the red line represents the probability for placebo

A probabilistic sensitivity analysis, conducted using 10,000 Monte Carlo simulations, further supported the robustness of the model findings (Fig. 3). At the exploratory threshold of three times GDP per capita ($96,981), eptinezumab was considered cost-effective in 98% of simulations. The cost-effectiveness acceptability curve demonstrated that eptinezumab became increasingly likely to be cost-effective at WTP thresholds above $75,000 per QALY (Fig. 4). These results highlight the high probability that eptinezumab could be considered a cost-effective option for migraine prevention in Taiwan under current economic assumptions.

## Discussion

This evaluation demonstrated that, over a six-month time horizon, eptinezumab provided an additional gain of 0.05 QALYs (0.35 vs. 0.31) and incurred an incremental cost of $3,396 ($4,461 vs. $1,065) compared with placebo, resulting in an ICER of $73,929 per QALY gained. When interpreted against GDP-based benchmarks for Taiwan, this ICER falls between one and three times GDP per capita. Sensitivity analyses confirmed the robustness of these findings, with the ICER remaining below the exploratory upper threshold in most scenarios, even when key parameters such as utilities associated with migraine frequency and drug costs were varied.

Our findings align with a scenario analysis from a recent United Kingdom study that used a discrete event simulation (DES) model from a societal perspective and also evaluated cost-effectiveness from the health system payer’s perspective (i.e., the National Health Service) [31]. That study reported an average gain of 0.231 QALYs and a cost saving of £7,655 over a five-year time horizon when comparing eptinezumab with best supportive care. The differences between the two studies can largely be explained by variations in model structure, time horizon, and data sources. For example, while our study used a Markov model with a six-month horizon to reflect dosing schedules and short-term treatment effects, Griffin et al. applied a DES framework to capture long-term disease progression. Differences in HRQoL measurement also contributed to the variations; the UK study used the Migraine-Specific Quality of Life questionnaire, whereas our analysis relied on utility estimates from an Asian cohort treated with fremanezumab. Furthermore, Griffin et al. incorporated a regression-based disutility function based on MMDs and treatment status, while our model used discrete utility values corresponding to MMD severity levels. Despite these methodological differences, both studies concluded that eptinezumab is a cost-effective strategy from the health payer’s perspective, reinforcing its clinical benefits across different modeling approaches and healthcare systems.

Although our analysis focused specifically on eptinezumab versus placebo, it is important to consider the broader landscape of CGRP inhibitors. Recent systematic reviews and network meta-analyses suggest similar efficacy across the CGRP inhibitor class (erenumab, galcanezumab, fremanezumab, and eptinezumab), with differences primarily in administration route, dosing frequency, and target specificity [32]. The intravenous administration of eptinezumab may offer advantages in terms of bioavailability and onset of action, with effects observed as early as 24 h after administration. However, these potential benefits must be weighed against the requirement for quarterly clinical visits. Therefore, the choice between CGRP inhibitors should be guided not only cost-effectiveness but also patient preferences, administration logistics, and healthcare system capacity.

A cost-effectiveness analysis conducted in the Netherlands from a healthcare payer perspective reported, in a scenario analysis excluding productivity loss, an ICER of €17,498 (approximately $20,286) per QALY gained for fremanezumab in the primary study population [33]. In Japan, an analysis estimated an ICER of ¥9,952,007 (approximately $67,738) per QALY gained for fremanezumab in both EM and CM populations [34]. Similarly, Sussman et al. reported an ICER of $122,167 per QALY for erenumab in patients with EM, while finding cost savings among those with CM in the United States [35]. Variations in ICER values across studies likely reflect differences in quality-of-life weights, patient characteristics, healthcare resource costs, and country-specific health system structures. Nevertheless, all evaluations consistently demonstrated substantial reductions in monthly migraine days, highlighting the clinical effectiveness of CGRP inhibitors, including eptinezumab, across diverse populations and healthcare systems.

Implementation considerations are crucial for evaluating the potential integration of eptinezumab into the Taiwanese healthcare system. Unlike other CGRP inhibitors that are self-administered subcutaneously, eptinezumab requires intravenous infusion every 12 weeks in a healthcare setting. This route of administration demands adequate infusion capacity within hospitals or outpatient clinics, trained healthcare personnel, and patient time for facility visits. In Taiwan’s National Health Insurance system, reimbursement policies for facility-administered medications differ from those for self-administered treatments, potentially affecting budget impact and patient access [36]. However, the quarterly administration schedule may enhance medication adherence compared with monthly self-injections, particularly among patients with adherence challenges or injection anxiety. These logistical and system-level factors, though beyond the scope of our economic model, influence the real-world adoption and cost-effectiveness of eptinezumab in Taiwan.

To the best of our knowledge, this is the first economic evaluation of eptinezumab for migraine prevention that incorporates Asian-specific parameters, including locally derived cost and utility estimates. This approach provides insights directly relevant to healthcare decision-making in Taiwan. Although the ICER was sensitive to changes in drug costs and utility values, probabilistic sensitivity analyses consistently supported the cost-effectiveness of eptinezumab across a wide range of plausible scenarios. By adopting a health payer’s perspective and focusing solely on direct medical costs, this study offers a targeted view of the economic burden of migraine within the healthcare financing framework. These strengths position the analysis as a valuable resource for policymakers seeking to optimize resource allocation for migraine prevention in Taiwan.

However, this study acknowledges several limitations. First, because eptinezumab-specific HRQoL data for Taiwanese patients were not available, the model used MSQ-derived EQ-5D-3 L utility values published by Wang et al., which were based on fremanezumab trials conducted in Japan and Korea [28]. These utilities demonstrated greater sensitivity to changes in monthly migraine days compared with raw EQ-5D-5 L values and were therefore considered more appropriate for capturing HRQoL impacts in migraine. Although sensitivity analyses indicated that the conclusions were robust to variations in utility values, future evaluations would benefit from locally derived HRQoL data to improve precision. Second, because mortality was not modeled, QALY differences reflected only changes in utility values. Sensitivity analyses indicated that utilities had the greatest influence on the ICER, underscoring the importance of HRQoL assumptions in cost-effectiveness studies of migraine. Although alternative utility sources or more extreme assumptions could produce different ICER estimates, the overall conclusions remained robust across plausible ranges.

Third, the model assumed stable transition probabilities across cycles, which may not fully capture real-world variations in treatment response. As this is a model-based analysis, results may not perfectly reflect real-world patterns. However, evidence from a recent randomized controlled trial conducted predominantly in an Asian population reported a treatment difference of − 2.4 MMDs for eptinezumab 100 mg versus placebo (− 7.2 vs. −4.8; *p* < 0.0001), which is comparable to the effect size estimated in our model [37]. This consistency suggests that the assumed reduction in MMDs is reasonable, and the findings are unlikely to be materially affected by this limitation.

Fourth, this analysis focused on a six-month time horizon, aligned with the PROMISE and DELIVER trials, to avoid uncertainty from extrapolating beyond trial-observed data. Although longer-term extension studies exist, the rationale for not extrapolating beyond the observed trial period is that these studies are limited by open-label design and lack randomized comparators, making long-term projections uncertain. Finally, a societal-perspective evaluation was not conducted because of the absence of reliable Taiwan-specific data on productivity loss, although such an analysis would likely demonstrate additional benefits due to reductions in work impairment and productivity loss.

## Conclusion

Eptinezumab offers meaningful health benefits compared with placebo in the prevention of migraine, resulting in notable QALY gains over a six-month period. When evaluated from a health insurance perspective, it was found to be a cost-effective option within the Taiwanese context. Although further research is warranted to confirm its long-term cost-effectiveness and to generate locally specific utility data, this study provides important early evidence to inform treatment decisions and guide healthcare resource allocation in Taiwan’s healthcare system.

## Supplementary Information

Supplementary Material 1.

## Data Availability

Data and the model will be made available on request.
