# Supplementary material for: Cost-utility analysis of eptinezumab for migraine prevention in Taiwan
Source: Health Econ Rev. 2025 Dec 28;16:11. doi: 10.1186/s13561-025-00711-x (PMC12860139; doi:10.1186/s13561-025-00711-x)
Supplement: Supplementary file 1 — Supplementary Material 1. [file 13561_2025_711_MOESM1_ESM.docx]

**Supplementary Methods**

***Age at entry***

Data from the PROMISE-1 (mean age: 40.0 years, standard deviation [SD]: 10.66 years), PROMISE-2 (mean age: 41.0 years, SD: 11.7 years), and DELIVER (mean age: 44.6 years, SD: 10.8 years) studies were used to derive the cumulative distribution function for age at study entry. Age sampling across the simulated cohort was assumed to follow a normal distribution (Figure S1).

| 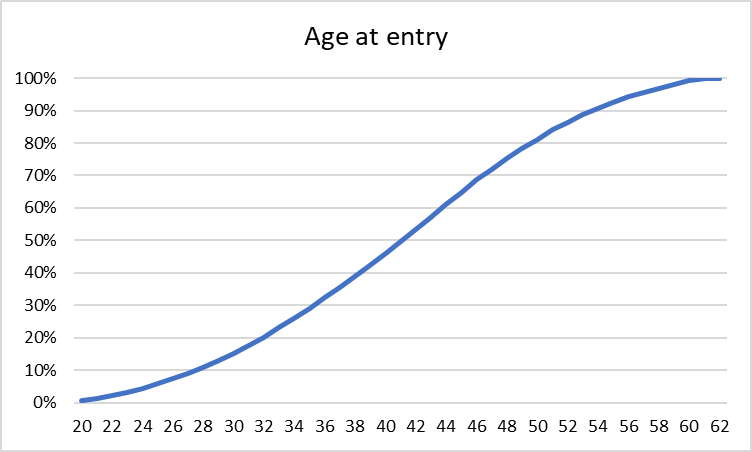 |
| --- |
| **Figure S1.** Normal distribution for sampling age at model entry |

***Baseline MMD values***

Baseline MMD frequencies were sampled for simulated individuals assigned to either EM or CM status. Data were derived from clinical trial reports of the PROMISE-1, PROMISE-2, and DELIVER studies. Beta distributions were fitted to the MMD parameters extracted from each trial, with resulting distributions characterized by mean (SD) values of 10 (3.02) for PROMISE-1, 20.4 (3.1) for PROMISE-2, and 14.5 (5.6) for DELIVER. Additionally, a beta distribution was fitted to the weighted average of MMD values from the three trials and included in the comparative analysis (Figure S2). Baseline MMD sampling was conducted independently of age and sex.

| 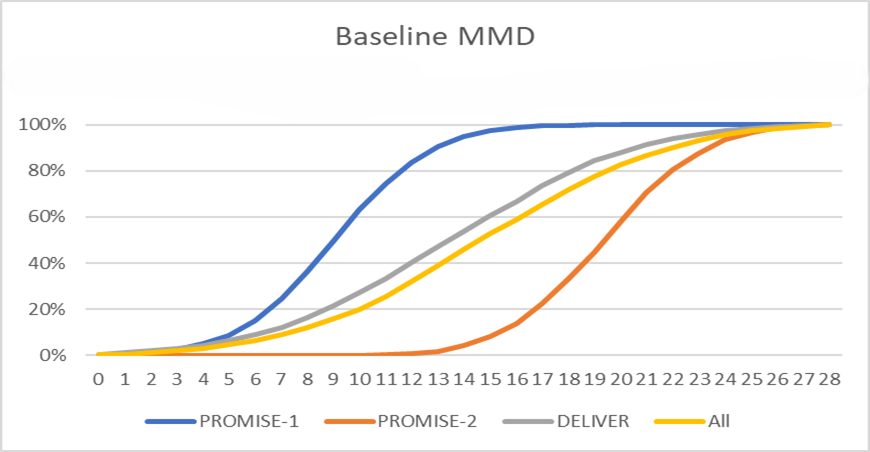 |
| --- |
| **Figure S2.** Beta distribution for sampling MMDs at baseline |

| **Supplementary Table 1.** Mean cost and utilization proportions for diagnostic procedures and treatments among patients with CM and EM | | | |
| --- | --- | --- | --- |
| **Description** | **Mean Cost (USD)** | **CM rates** | **EM rates** |
| MRI (without contrast) | 195.9 | 28.70% | 20.70% |
| CT (without contrast) | 114.5 | 28.70% | 20.70% |
| ECG | 4.5 | 28.70% | 20.70% |
| Skull X-ray film (including each view of skull film) | 6.0 | 28.70% | 20.70% |
| Blood test | 6.0 | 75.00% | 55.90% |
| Botulinum toxin injection for CM | 110.4 | 6.40% | 0.00% |
| Transcutaneous electrical nerve stimulator | 9.6 | 0.60% | 0.00% |
| Peripheral nerve block | 120.5 | 0.60% | 0.00% |
| Abbreviations: MRI – magnetic resonance imaging; CM – chronic migraine; CT – computed tomography; ECG – electrocardiogram, EM – episodic migraine | | | |
